# Acute Electrocardiographic Changes Coincident With Dofetilide Overdose and Acute Coronary Syndrome

**DOI:** 10.1016/j.jaccas.2024.102709

**Published:** 2024-11-20

**Authors:** David J. King, Mudit Dutta, Ramil Goel, Kun Xiang

**Affiliations:** a Department of Medicine, University of Florida, Gainesville, Florida, USA; b VAMC and the University of Florida, Division of Cardiovascular Medicine, Gainesville, Florida, USA; c Division of Cardiovascular Medicine, University of Florida, Gainesville, Florida, USA

**Keywords:** acute coronary syndrome, dofetilide, I_KR_ potassium channel, I_Na_ sodium channel, non-ST-segment elevation myocardial infarction, NSTEMI, QT prolongation, Tikosyn

## Abstract

A 73-year-old man with atrial fibrillation and coronary disease requiring stenting to the right coronary artery 7 months prior was admitted for observation after taking an extra dofetilide dose. Troponins trended upward, and electrocardiogram demonstrated QT prolongation to 502 ms as well as T-wave inversions. The patient underwent cardiac catheterization, which revealed severe distal left main disease.

## Graphical Abstract

**Figure:**
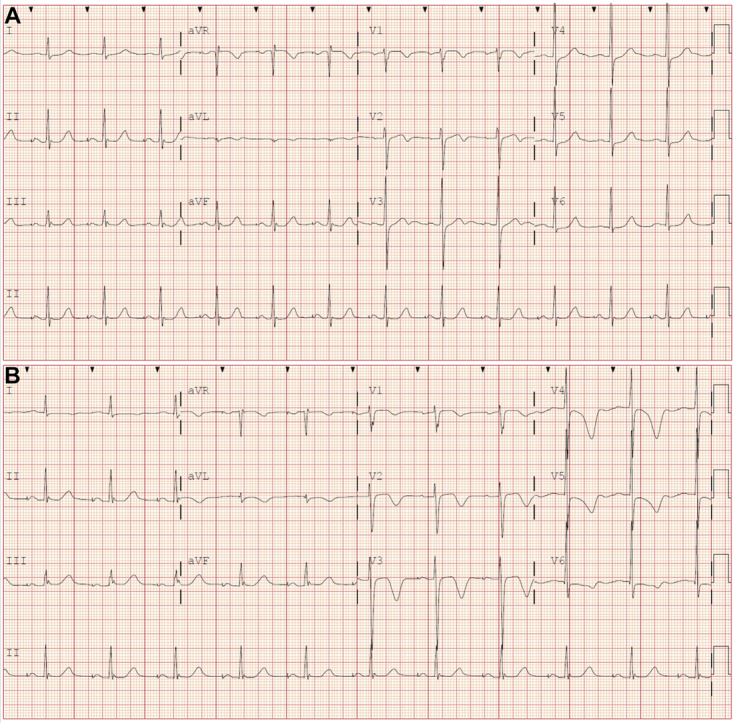

## History of Presentation

A 73-year-old man presented with anxiety and “heartburn” after taking an extra dose of dofetilide 500 μg. History included atrial fibrillation on apixaban and dofetilide, sick sinus syndrome requiring dual-chamber pacemaker, gastroesophageal reflux, and coronary disease with stenting to a 99% lesion of the right coronary artery 7 months before admission. Previous catheterization showed 50% left main stenosis, 30% left anterior descending stenosis, and 80% first diagonal stenosis that did not require intervention. He denied alcohol, tobacco, and illicit substance use. On examination, the patient had an anxious affect without cardiopulmonary abnormalities.

**Take-Home Messages**

• Dofetilide affects the QT interval through modification of the I_KR_ potassium channel, while NSTEMI does so via the I_Na_ sodium channel.
• It is important to maintain a broad differential diagnosis when determining the cause of QT prolongation.

## Investigations

**Figure 1:**
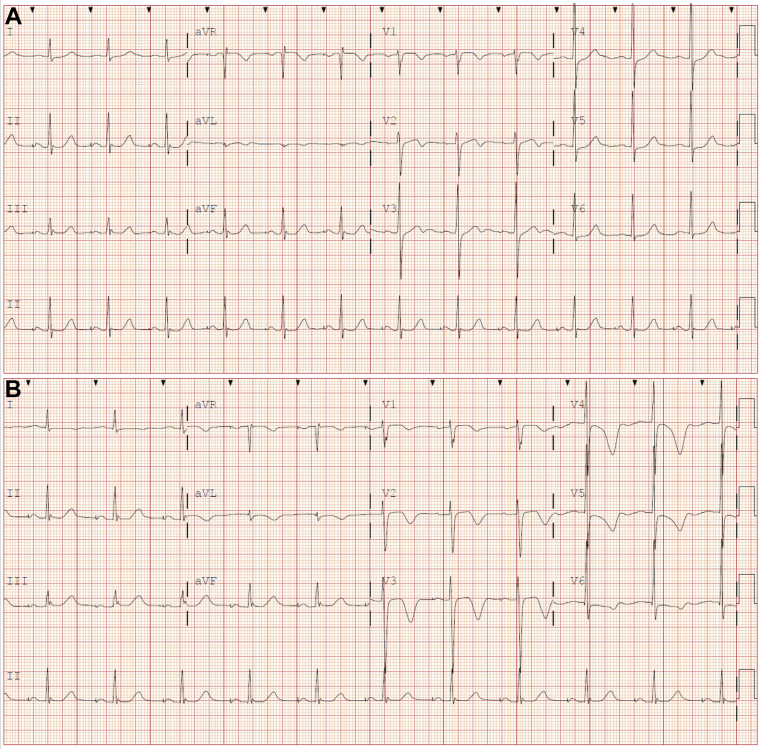
Presenting and Follow-Up ECGs (A) Presenting electrocardiogram (ECG) demonstrating an atrial-paced ventricular-sensed rhythm, normal QTc, and anteroseptal T-wave abnormalities. (B) Repeat ECG with anterolateral T-wave inversions and QT prolongation to 502 ms.

Electrocardiography (ECG) showed an atrial-paced ventricular-sensed rhythm at 72 beats/min with normal QT interval (Figure 1A). High-sensitivity troponin I was 18 pg/mL (upper reference limit = 15 pg/mL), with unremarkable metabolic panel and complete blood count. The patient was admitted for QT interval observation following dofetilide overdose. He continued to experience “heartburn” and received pantoprazole. Repeat ECG demonstrated QT prolongation to 502 ms, precordial T-wave inversions (Figure 1B), and repeat troponin was 108 pg/mL.

What is the most appropriate next step in management?

A) Consent the patient for cardiac catheterization
B) Contact poison control and pursue gastric lavage
C) Inpatient pharmacologic stress testing
D) Neurology consultation given development of neurogenic T waves
E) Watchful waiting strategy for dofetilide washout

## Discussion

The correct answer is A. The patient had “heartburn,” ECG changes, and uptrending troponins suggesting acute non-ST-segment elevation myocardial infarction. Subsequent urgent cardiac catheterization revealed severe distal left main disease. Answers B and C would be appropriate for intermediate-risk patients, but this presentation should raise suspicion for acute coronary syndrome (ACS) as opposed to dofetilide overdose alone. Watchful waiting (E) would have missed the true culprit of his symptoms. Last, the patient had no evidence of a neurologic deficit to prompt consultation (D).

In ACS, QT prolongation is considered the earliest sign of myocardial ischemia.^1^ This ECG abnormality is due to modification of the inward sodium current (I_Na_), increasing action potential duration and prolonging repolarization.^1^ QT prolongation from dofetilide use is secondary to its effect on the rapid delayed outward rectifier potassium current (I_KR_); decreased potassium efflux increases time taken by the myocyte to reach resting membrane potential, slowing repolarization.^2^^,^^3^ Causes of QT prolongation include congenital channelopathies, left ventricular hypertrophy, hypokalemia, hypomagnesemia, hypocalcemia, and numerous medications. Although perturbation of the I_KR_ potassium channel is the most common cause of acquired QT prolongation—the mechanism shared by most QT-prolonging medications^3^—it was not the etiology of this patient’s presentation. The case highlights that early ACS can present with QT prolongation, and that dofetilide use could mask interpretation.

## Funding Support and Author Disclosures

The authors have reported that they have no relationships relevant to the contents of this paper to disclose.
